# 

**Published:** 1885-06

**Authors:** 


					﻿Vhole Number, 306.
JUNE, 1885.
Vol. L., Number 6.
THE
CHICAGO MEDICAL JOURNAL AND EXAMINER
EDITORS:
S. J. JONES, M.D., LL.D.
W. W. JAGGARD, A.M., M.D
HAROLD N. MOYER, M.D.
CHICAGO:
PUBLISHED MONTHLY BY THE CHICAGO MEDICAL PRESS ASSOCIATION,
240 Wabash Avenue.
CLARK & LONGLEY, PRINTERS, 163 AND 165 DEARBORN STREET, CHICAGO.
				

